# Neurologic Disease after Yellow Fever Vaccination, São Paulo, Brazil, 2017–2018

**DOI:** 10.3201/eid2706.204170

**Published:** 2021-06

**Authors:** Ana Freitas Ribeiro, Bruno Fukelmann Guedes, Jamal M.A.H. Sulleiman, Francisco Tomaz Meneses de Oliveira, Izabel Oliva Marcilio de Souza, Juliana Silva Nogueira, Rosa Maria Nascimento Marcusso, Eder Gatti Fernandes, Guilherme Sciascia do Olival, Pedro Henrique Fonseca Moreira de Figueiredo, Ana Paula Rocha Veiga, Flávia Esper Dahy, Natália Nasser Ximenes, Lecio Figueira Pinto, José Ernesto Vidal, Augusto Cesar Penalva de Oliveira

**Affiliations:** Universidade Nove de Julho, São Paulo, Brazil (A.F. Ribeiro);; Instituto de Infectologia Emílio Ribas, São Paulo (A.F. Ribeiro, J.M.A.H. Sulleiman, R.M. Nascimento Marcusso, G. Sciascia do Olival, A.P. Rocha Veiga, F. Esper Dahy, J.E. Vidal, A.C. Penalva de Oliveira);; Hospital das Clínicas, Universidade de São Paulo, São Paulo (B.F. Guedes, I.O. Marcilio de Souza, P.H.F. Moreira de Figueiredo, N. Nasser Ximenes, L. Figueira Pinto, J. Ernesto Vidal);; Irmandade Santa Casa de Misericórdia de São Paulo, São Paulo (F.T. Meneses de Oliveira);; Instituto Adolfo Lutz, São Paulo (J. Silva Nogueira);; Centro de Vigilância Epidemiológica Prof. Alexandre Vranjac, São Paulo (E. Gatti Fernandes)

**Keywords:** encephalitis, aseptic meningitis, Guillain-Barré syndrome, myelitis, optic neuritis, autoimmune encephalitis, yellow fever, vaccine, viruses, vaccine-associated adverse events, São Paulo, Brazil, meningitis/encephalitis

## Abstract

Yellow fever (YF) vaccine can cause neurologic complications. We examined YF vaccine–associated neurologic disease reported from 3 tertiary referral centers in São Paulo, Brazil, during 2017–2018 and compared the performance of criteria established by the Yellow Fever Vaccine Working Group/Centers for Disease Control and Prevention and the Brighton Collaboration. Among 50 patients who met inclusion criteria, 32 had meningoencephalitis (14 with reactive YF IgM in cerebrospinal fluid), 2 died, and 1 may have transmitted infection to an infant through breast milk. Of 7 cases of autoimmune neurologic disease after YF vaccination, 2 were acute disseminated encephalomyelitis, 2 myelitis, and 3 Guillain-Barré syndrome. Neurologic disease can follow fractional vaccine doses, and novel potential vaccine-associated syndromes include autoimmune encephalitis, opsoclonus-myoclonus-ataxia syndrome, optic neuritis, and ataxia. Although the Brighton Collaboration criteria lack direct vaccine causal assessment, they are more inclusive than the Centers for Disease Control and Prevention criteria.

## Abstract 2

Disease should be diagnosed by using the Brighton Collaboration case definitions and cerebrospinal fluid IgM reactivity.

Yellow fever (YF) is an acute febrile illness caused by a mosquito-borne arbovirus of the family *Flaviviridae*. The disease is endemic to the tropical forests of South America and Africa, periodically causing outbreaks and epidemics. The clinical manifestations of YF range from asymptomatic to severe with jaundice and hemorrhage (*1*). The primary preventive strategy is vaccination. The 3 substrains of the 17D vaccine virus currently used for vaccine production (17DD, 17D-204, and 17D-213) have similar safety and immunogenicity profiles (*1*,*2*). The main YF vaccine available in Brazil is 17DD, which is produced by Bio-Manguinhos-Fiocruz (https://www.bio.fiocruz.br). YF vaccine–associated neurologic disease (YEL-AND) is a rare but potentially severe adverse event following immunization (AEFI). The incidence of YEL-AND varies between studies; in the United States and Brazil, the estimated range is 0.2–0.94 cases/100,000 doses (*3*–*6*).

In 2002, the Centers for Disease Control and Prevention (CDC) formed the Yellow Fever Vaccine Safety Working Group, a panel of vaccine safety experts, which proposed a surveillance case definition for YEL-AND. The clinical manifestations included in YEL-AND are meningoencephalitis (neurotropic disease), Guillain-Barré syndrome (GBS), and acute disseminated encephalomyelitis (ADEM) (*7*).

In 2004, the Brighton Collaboration (BC) was commissioned as a vaccine safety research network to develop standardized case definitions for AEFI (*8*). The first BC case definition of aseptic meningitis was issued in 2007 (*9*). Subsequent BC criteria were established for encephalitis, ADEM (*10*), and myelitis (*10*), all distinct from aseptic meningitis (*9*) and each other.

There are fundamental differences between the BC and CDC case definitions. The CDC criteria require that acute brain lesions or dysfunction be evidenced by electroencephalography (EEG) or magnetic resonance imaging (MRI) and exclude causality when the vaccine–symptom interval exceeds 30 days. These criteria render them poorly suited to diagnose YEL-AND in resource-limited settings or during massive vaccination campaigns. The BC criteria encompass a broader range of neurologic syndromes, including aseptic meningitis and myelitis. However, in contrast to the CDC criteria, they lack specific criteria to determine vaccine causality (YF virus IgM in cerebrospinal fluid [CSF]). Both criteria focus on major neurologic syndromes and overlook the rare and atypical ones. Although recent publications used the newer BC criteria (*6*), CDC case definitions are still used routinely by the Brazil Ministry of Health, as seen in the Epidemiologic Surveillance of Post-Vaccination Adverse Events manual (*11*).

During 2017 and 2018, YF virus transmission increased in the southeastern region of Brazil (states of Rio de Janeiro, Espírito Santo, and those parts of São Paulo where the vaccination schedule did not include YF vaccine). In response to this outbreak, the National Immunization Program launched a massive vaccination campaign in the São Paulo metropolitan area. During 2017–2018, a total of 6 million full doses (0.5 mL) and 4 million fractional doses (0.1 mL) of 17DD were administered throughout the São Paulo metropolitan area (E. Gatti Fernandes, unpub. data). We describe suspected YEL-AND cases from tertiary centers in the city of São Paulo during the 2017–2018 vaccination campaign, identify differences between the CDC and BC classification criteria, and describe novel atypical syndromes.

## Methods

Our retrospective study included cases from 3 tertiary referral hospitals in the city of São Paulo (Hospital das Clínicas da Faculdade de Medicina da USP, Instituto de Infectologia Emilio Ribas, and Santa Casa de Misericórdia de São Paulo). We included patients who had been vaccinated during the campaign and for whom a case of suspected YEL-AND was reported to the National Post-Vaccination Adverse Events Surveillance System; for patients with nonnotified cases, we included those whose attending physician recognized the case as potential YEL-AND. All cases were included in the initial analysis, regardless of vaccine–symptom interval. We reviewed the YF vaccination information (first or booster dose, full or fractional dose, alone or in combination with other vaccines); clinical, epidemiologic, and laboratory data from electronic charts; laboratory databases; and (when available) a structured AEFI notification form.

We first classified and analyzed all cases according to the BC criteria for the diagnosis of aseptic meningitis (*9*), encephalitis, myelitis, ADEM (*10*), and GBS (*12*) (Appendix 1). We excluded from final analysis patients with alternative diagnoses or insufficient information. Neurologic autoimmune diseases were not excluded when the YF vaccine was biologically plausible as a trigger. When these atypical clinical syndromes were identified, we assessed causality by using a tool proposed by the World Health Organization (*13*). To compare the performance of the different classification criteria, we also classified cases according to the Brazil Ministry of Health manual (*11*), (Appendix 2), using the same exclusion criteria as the BC criteria. Difficult diagnoses were decided at consensus meetings.

All analyses were performed by using R statistics software version 3.6.3 (https://www.r-project.org). Significance was set at p<0.05 for all statistical comparisons.

## Results

We identified 50 suspected YEL-AND cases at the 3 tertiary care facilities. Of these, we excluded 8 (16%) cases, 3 because of insufficient information and 5 because of alternative diagnoses (1 each of GBS and Zika virus–reactive IgM in CSF, neurosurgery-associated bacterial meningitis, multiple sclerosis preceding vaccination and postvaccination demyelination, mononucleosis-like syndrome with acute toxoplasmosis, and meningoencephalitis with a positive rapid test result for dengue virus [DENV]).

The final analysis included 42 patients 1–89 years of age; most were male (62%) and White (74%). The median time between vaccination and symptom onset was 15 days (interquartile range [IQR] 5.5–20.0). Cases were associated with the first dose of the YF vaccine for 28 patients and with booster doses for 2 patients; this information was missing for 12 patients. A total of 9 patients received fractional doses and 30 received full doses; this information was missing for 3 patients. For all patients, the YF vaccine was given alone. All patients underwent CSF examination. YF virus IgM immunoreactivity in CSF was performed for 30 patients; reactivity was detected for 15. Reverse transcription PCR for YF virus was performed on CSF for 28 patients; all results were negative. Testing for DENV IgM was also performed on CSF of 28 patients; all results were negative (Tables 1, 2; Appendix 3).

**Table 1:** Diagnostic certainty, clinical, epidemiologic, and immunologic investigations for 42 patients with suspected yellow fever vaccine–associated neurologic disease, according to Brighton Collaboration classification criteria, São Paulo, Brazil, 2017–2018*

Characteristic	Aseptic meningitis, n = 24	Encephalitis, n = 8	Guillain-Barré syndrome, n = 3‡	Myelitis, n = 2‡	ADEM, n = 2‡	Unclassified† n = 3‡
Age, y, median (IQR) [range]	36 (23.75–46.75)	40 (30.25–58.25)	59 (43–73)	33 [25–41]	37 [22–52]	28 [25–50]
Sex, no (%)						
F	7 (29)	5 (62)	1 (33)	2 (100)	1 (50)	0
M	17 (71)	3 (38)	2 (67)	0	1 (50)	3 (100)
Vaccine–symptom interval, d, median (IQR) [range]	17 (7.75–20.00)	7 (3.50–17.25)	16 [14–31]	11.5 [0–23]	10 [5–15]	13 [3–29]
No. full/fractional/unknown doses	18/4/2	5/3/0	3/0/0	1/1/0	1/1/0	2/0/1
YF virus IgM in CSF, reactive/total tested	10/17	4/7	0/2	0/1	1/2	0/1
YF virus in CSF detected by PCR, detected/total tested	0/17	0/6	0/2	0/1	0/1	0/1
BC level of diagnostic certainty, no. cases						
Level 1	17	0	1	0	2	NA
Level 2	7	8	2	2	0	NA
Brazil MoH/CDC classification, no. cases	Level 1 NRL: 21; level 2 NRT: 1; definite NRT: 1; suspected NRT: 1	Level 1 NRL: 2; level 2 NRT: 3; definite NRT: 2; suspected NRT: 1	Level 2 PNS: 1; probable PNS:2	Level 1 NRL: 2	Probable CNS: 2	Level 1 NRL: 3

*ADEM, acute disseminated encephalomyelitis; BC, Brighton Collaboration; CDC, Centers for Disease Control and Prevention); CNS, autoimmune neurologic disease with central nervous system involvement; CSF, cerebrospinal fluid; IQR, interquartile range; NA, not applicable; NRL, neurologic disease; NRT, neurotropic disease; MoH, Ministry of Health; PNS, autoimmune neurologic disease with peripheral nervous system involvement; YF, yellow fever. †Includes 1 case of ataxia, 1 of opsoclonus-myoclonus-ataxia, 1 case of optic neuritis. ‡In groups with <5 cases, range is substituted for IQR.

**Table 2:** Laboratory, neurophysiologic, and imaging characteristics for 42 patients with suspect YEL-AND, according to classification with the Brighton Collaboration criteria, São Paulo, Brazil, 2017–2018*

Variable	Aseptic meningitis, n = 24	Encephalitis, n = 8	Guillain-Barré syndrome, n = 3‡	Myelitis, n = 2‡	ADEM, n = 2‡	Unclassified,† n = 3‡
CSF parameters§						
Leukocytes >5, no. (%)	24 (100)	7 (87.5)	1 (33)	0	1 (50)	1 (33)
Leukocytes, total/mm^3^, median (IQR) [range]	76.50 (53–207.5)	30 (13–70)	1 [0–32]	1 [0–2]	4.5 [2–7]	2 [1–12]
Lymphocytes, median (IQR) [range]	73 (65.5–88.0)	85 (71–93)	51.5 [3 –71]	75 [75–75]	79,5 [79–80]	80 [73–92]
Neutrophils, median (IQR) [range]	10 (3.5–25.0)	3 (0.5–6.0)	23.5 [13–34]	16 [16–16]	19.5 [19–20]	2 [1–3]
Erythrocytes, total/mm^3^, median (IQR) [range]	2 (1–12)	5.5 (1–640.50)	302 [249–355]	26 [1–52]	985.5 [131–1,840]	0 [0–3]
Total protein, mg/dL, median (IQR) [range]	53.5 (48–71.5)	60 (47.5–67)	53 [31–66]	27.5 [23–32]	61 [41 – 81]	46 [26–51]
Total glucose, mg/dL, median (IQR) [range]	60 (52.5–64.5)	66 (54.5–92.5)	60.5 [50–71]	72 [66–78]	62.5 [54–71]	
MRI findings, no. cases	Leptomeningeal enhancement, 1; unremarkable, 3	Leptomeningeal enhancement, 1; unremarkable, 5	Facial nerve enhancement, 1; unremarkable, 1	Longitudinally extensive myelitis,1; partial myelitis, 1	White matter abnormalities and extensive myelitis,1; brainstem and cerebellar peduncles abnormalities, 1	Bilateral optic nerve abnormalities,1; unremarkable, 1
EEG/EMG findings, no. cases	EEG: disorganized background, 2; unremarkable, 2	EEG: disorganized background, 6	EMG: AMAN, 1	ND	ND	ND

*ADEM, acute disseminated encephalomyelitis; AMAN, axonal motor polyneuropathy; CSF, cerebrospinal fluid; EEG, electroencephalography; EMG, electromyography; IQR, interquartile range; ND, not done; YF, yellow fever. †Includes 1 case of ataxia, 1 of opsoclonus-myoclonus-ataxia, and 1 of optic neuritis. ‡In groups with <5 cases, range was substituted for IQR. §All patients underwent lumbar puncture and CSF analysis.

### Aseptic Meningitis

Twenty-four cases were classified as aseptic meningitis (diagnostic certainty level 1 or level 2). These patients had headaches (100%) and fever (92%), which developed a median of 17 days (IQR 7.75–20.0 days) after vaccination. CSF analysis showed a median of 76.5 leukocytes/mm^3^. YF virus IgM reactivity in CSF confirmed a vaccine-related disease for 10 patients. Attributing the disease to the YF vaccine was not possible for 14 other patients (nonreactive or unknown YF virus IgM), although for all 24 patients, a structured assessment with a tool proposed by the World Health Organization (*13*) suggested a causal association with vaccination. For IgM-reactive and IgM-nonreactive patients, no differences in CSF cell count or time from vaccination to symptom onset were noted. Except for 1 patient who died, the course of disease for these patients was uncomplicated, and they were discharged a median of 2.5 days after hospital admission (IQR 1.00–5.25 days). One case involved potential transmission through breast-feeding (atypical case).

### Encephalitis

Eight patients had encephalitis. Compared with those with aseptic meningitis, these patients had more seizures (50% vs. 0; p = 0.002), more psychosis (37.5% vs. 0; p = 0.011), and longer hospital stays (median 17.5 [IQR 12.00–35.25] vs. 2.5 [IQR 1.00–5.25) days; p = 0.002). One patient died, 4 displayed YF virus IgM reactivity in CSF, and 3 had autoimmune encephalitis.

### Autoimmune Disease: ADEM, Myelitis, and GBS

We identified 2 ADEM cases. For 1 patient, paraparesis and somnolence developed 15 days after YF vaccine, and MRI revealed diffuse demyelinating lesions in the brain and cervical spinal cord. This patient was the only one outside of the meningitis and encephalitis groups with CSF positive for YF virus IgM. For the other patient, ataxia developed 5 days after vaccination, and MRI showed T2-weighted and FLAIR signal abnormalities in the dorsal pons and middle cerebellar peduncles. That patient was not tested for YF virus antibodies or RNA.

Two patients experienced spastic quadriparesis, 20 hours and 23 days after vaccination. MRI analysis revealed partial myelitis in the cervical cord (first patient) and longitudinally extensive cervicothoracic myelitis (second patient). Three patients experienced flaccid quadriparesis (14, 16, and 31 days after vaccination) consistent with GBS. One patient underwent a nerve conduction study, which revealed axonal motor polyneuropathy.

### Atypical Cases

We found several cases of neurologic syndromes that are typically autoimmune or occur after infection but that are not traditionally associated with YF vaccination. The encephalitis group included 3 patients with autoimmune encephalitis and antibodies against neural targets. The first patient, a 42-year-old woman, experienced headache and fever 1 day after the first (fractioned) dose of the 17-DD vaccine, followed by psychosis and status epilepticus. She had altered EEG findings and inflammatory CSF; YF virus IgM in CSF was nonreactive. Antineurexin3 IgG was detected in serum and CSF. The second patient, a 14-year-old girl, experienced headache, depression, psychosis, seizures, and EEG slowing 21 days after receiving her first (full) dose of the 17-DD vaccine. A bloody CSF sample (1,142 erythrocytes/mm^3^) was reactive for YF virus IgM 3 months after symptom onset, although PCR for YF virus in CSF was negative. That result could be a false positive. The third patient, a 39-year-old woman, experienced fever, vertigo, and psychiatric symptoms 23 days after YF vaccination (full dose, first ever). She was evaluated by a neurologist 45 days after symptom onset. Examination showed opsoclonus-myoclonus-ataxia and encephalopathy, EEG revealed background slowing, and CSF (slightly bloody from a traumatic lumbar puncture) showed 5 leukocytes/mm^3^. Immunologic tests for YF virus were not performed in serum or CSF. N-methyl-D-aspartate receptor (NMDA-r) IgG was identified in serum and CSF of the second and third patients. These 3 cases are described in greater detail elsewhere (*14*). All 3 cases met the BC encephalitis case definition and the CDC criteria for level 2 neurotropic disease. However, because the CDC criteria require no evidence of other diagnoses, they were not further classified as suspected or probable YEL-AND.

Three patients exhibited autoimmune syndromes that are unclassifiable per both CDC and BC criteria. The first patient was a 25-year-old man in whom cerebellar ataxia, opsoclonus, and generalized myoclonus, consistent with opsoclonus-myoclonus-ataxia syndrome, developed 29 days after vaccination. MRI and EEG findings were unremarkable, and he recovered over a few months with immunotherapy. He was not investigated for YF virus–specific antibodies or nucleic acid. Information on vaccine (dosing, first, or booster dose) was missing. The second patient was a 50-year-old man in whom dysarthria, imbalance, and mild somnolence developed 13 days after he had received a first (full) dose of the 17-DD vaccine. Physical examination showed global cerebellar ataxia. Neuroimaging and CSF analysis were unremarkable, and the patient improved spontaneously over a few days. PCR and assays to detect YF virus IgM in CSF were not performed. The third patient was a 28-year-old man who reported frontal headache associated with eye movement, followed within 2 weeks by bilateral vision impairment. At admission, he had low visual acuity in the left eye, and MRI showed extensive signal abnormalities in both optic nerves, which was consistent with optic neuritis. CSF was inflammatory but negative for YF virus IgM or by PCR for YF virus. He recovered with immune therapy.

A fourth case occurred after the mother of a 1-year-old boy received her first dose of the YF vaccine (no information on dosing) but continued to breast-feed her child. Seven days after the mother’s vaccination, the infant exhibited nasal discharge, headache, fever, anorexia, and malaise. Examination indicated that he was alert and active but dehydrated. Computed tomography (CT) of the brain showed no abnormalities, and CSF analysis indicated 230 leukocytes/mm^3^, 12 erythrocytes/mm^3^, and 35 mg/dL protein. YF virus IgM and PCRs were not performed for infant or mother. The infant was discharged 9 days after admission.

### Fatal Cases

Two patients died. Aseptic meningitis developed in 1 and encephalitis in the other.

A 52-year-old woman with a history of underlying unruptured giant intracranial aneurisms experienced retro-orbital headache, fever, nausea, and vomiting 9 days after receiving a full dose of the YF vaccine. Examination showed nuchal rigidity but was otherwise unremarkable. Brain CT showed giant intracranial aneurysms without bleeding. A lumbar puncture revealed xanthochromic CSF with 3,080 leukocytes/mm^3^ (68% neutrophils, 15% lymphocytes), 2 erythrocytes/mm^3^, and 163 mg/dL protein. The woman was admitted to the intensive care unit (ICU), where she received treatment for presumed bacterial meningitis. CSF analysis was reactive for YF virus IgM, negative for YF virus by PCR, and negative for DENV IgM. On hospitalization day 14, seizures, left hemiplegia, and coma developed. A second CT showed focal brain edema and a malignant right middle cerebral artery stroke. She died 3 months later. Although the timing of symptoms, fever, the initially benign presentation, and reactive CSF IgM initially indicated a case of neurotropic disease, the underlying intracranial aneurysms, CSF xanthochromia, and cerebral infarction suggest subarachnoid hemorrhage as a relevant differential diagnosis. The 2 conditions may have occurred concurrently, and for this patient, it would be difficult to determine whether subarachnoid hemorrhage preceded or followed neurotropic disease.

A 19-year-old woman experienced myalgia, vomiting, and progressive headache that started 4 days after receiving a full dose of the 17DD vaccine alone. Mild confusion progressed steadily over the next 12 days. On postvaccination day 16, bilateral convulsive seizures developed; the woman was admitted to the ICU and was comatose at the time of arrival. Brain CT findings were unremarkable. Initial blood chemistry revealed elevated alanine (276U/L) and aspartate (246 U/L) aminotransferase levels (suggesting viscerotropic disease), but results were otherwise unremarkable. A lumbar puncture sample contained 19 leukocytes/mm^3^ and 154 mg/dL protein and was negative for YF virus IgM and nucleic acid. Over the next 14 days, sepsis, renal insufficiency, and disseminated intravascular coagulation developed, and the patient died. Autopsy detected centrilobular necrosis and periportal inflammation of the liver and revealed mild perivascular edema and congestion of brain sections. RNA extracted from formalin-fixed paraffin-embedded tissues was positive for YF virus in the lungs and heart but negative in the brain, spleen, and kidney. Because of the low quality of RNA, it was not possible to differentiate between wild type and vaccine strains. This patient experienced multiorgan failure later than usual for viscerotropic disease.

### Comparison of BC and CDC (and Brazil Ministry of Health) Classifications

**Figure 1:**
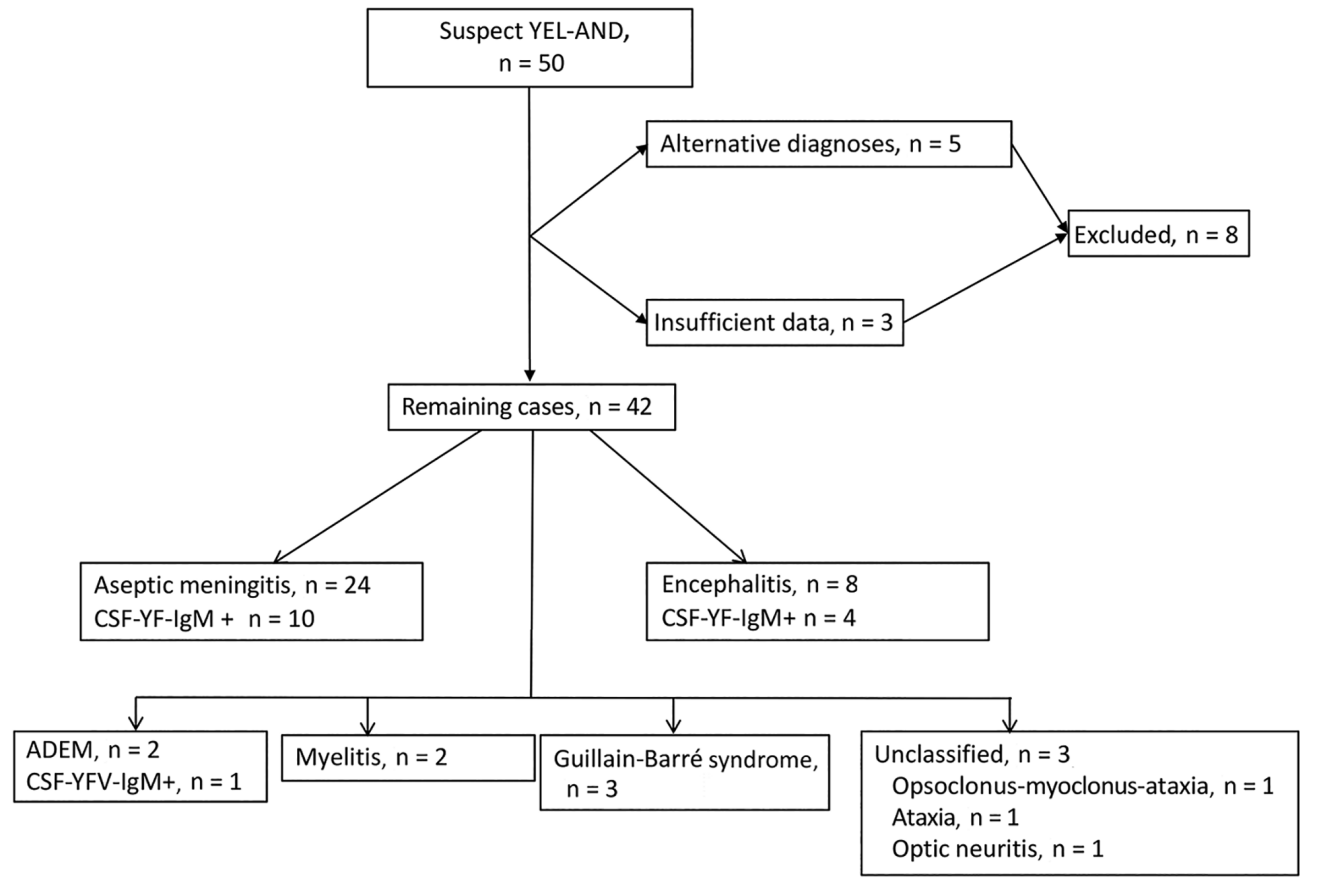
Classification of cases of yellow fever vaccine–associated neurologic disease with Brighton Collaboration criteria, São Paulo, Brazil, 2017–2018. CSF YF IgM, yellow fever virus IgM in cerebrospinal fluid; YEL-AND, yellow fever vaccine-associated neurologic disease; YF, yellow fever; +, positive.

**Figure 2:**
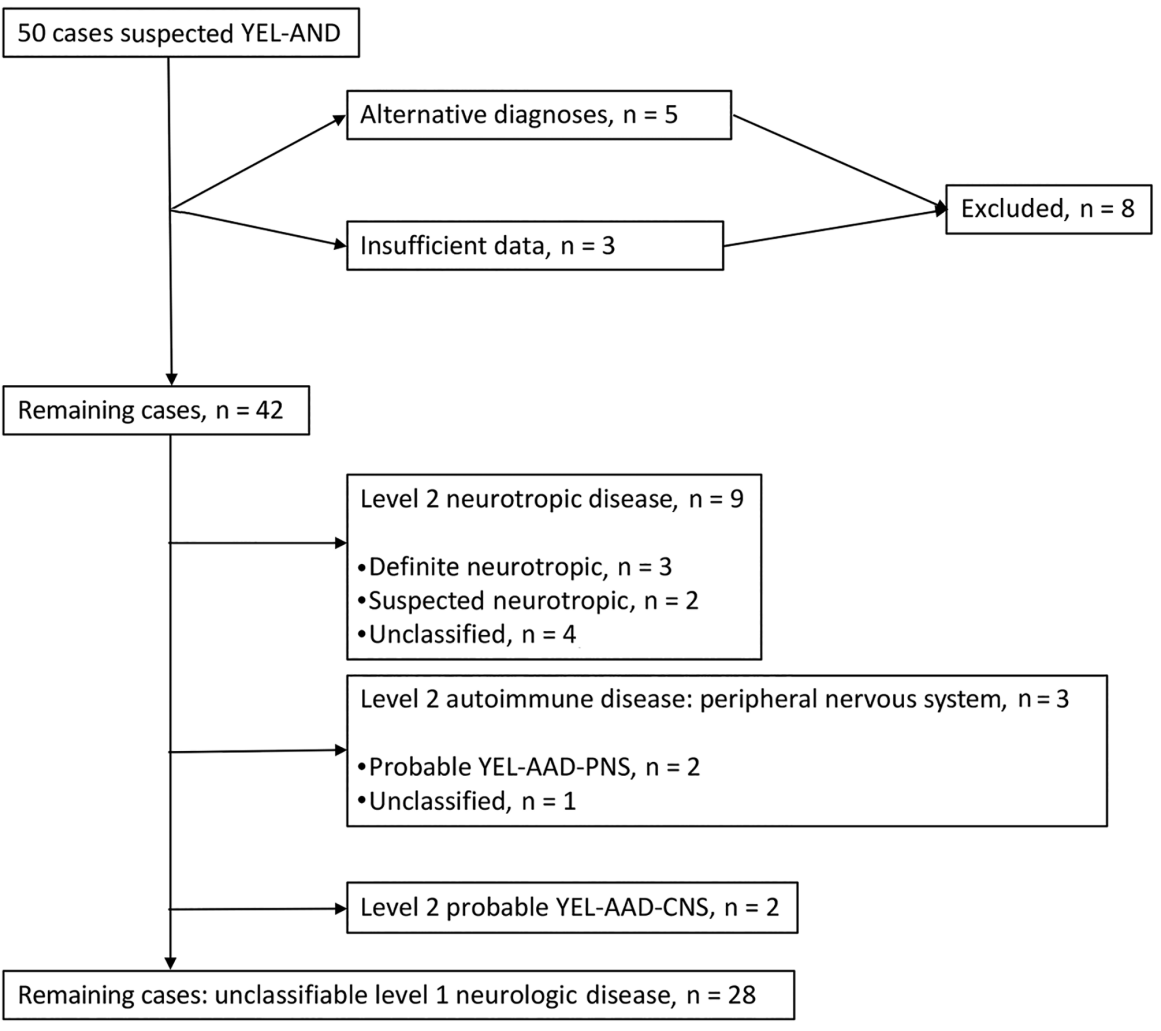
Classification of cases of yellow fever vaccine–associated neurologic disease with Centers for Disease Control and Prevention criteria, São Paulo, Brazil, 2017–2018. YEL-AAD-PNS, autoimmune disease with peripheral nervous system involvement; YEL-AAD-CNS, autoimmune disease with central nervous system involvement; YEL-AND, yellow fever vaccine-associated neurologic disease.

**Figure 3:**
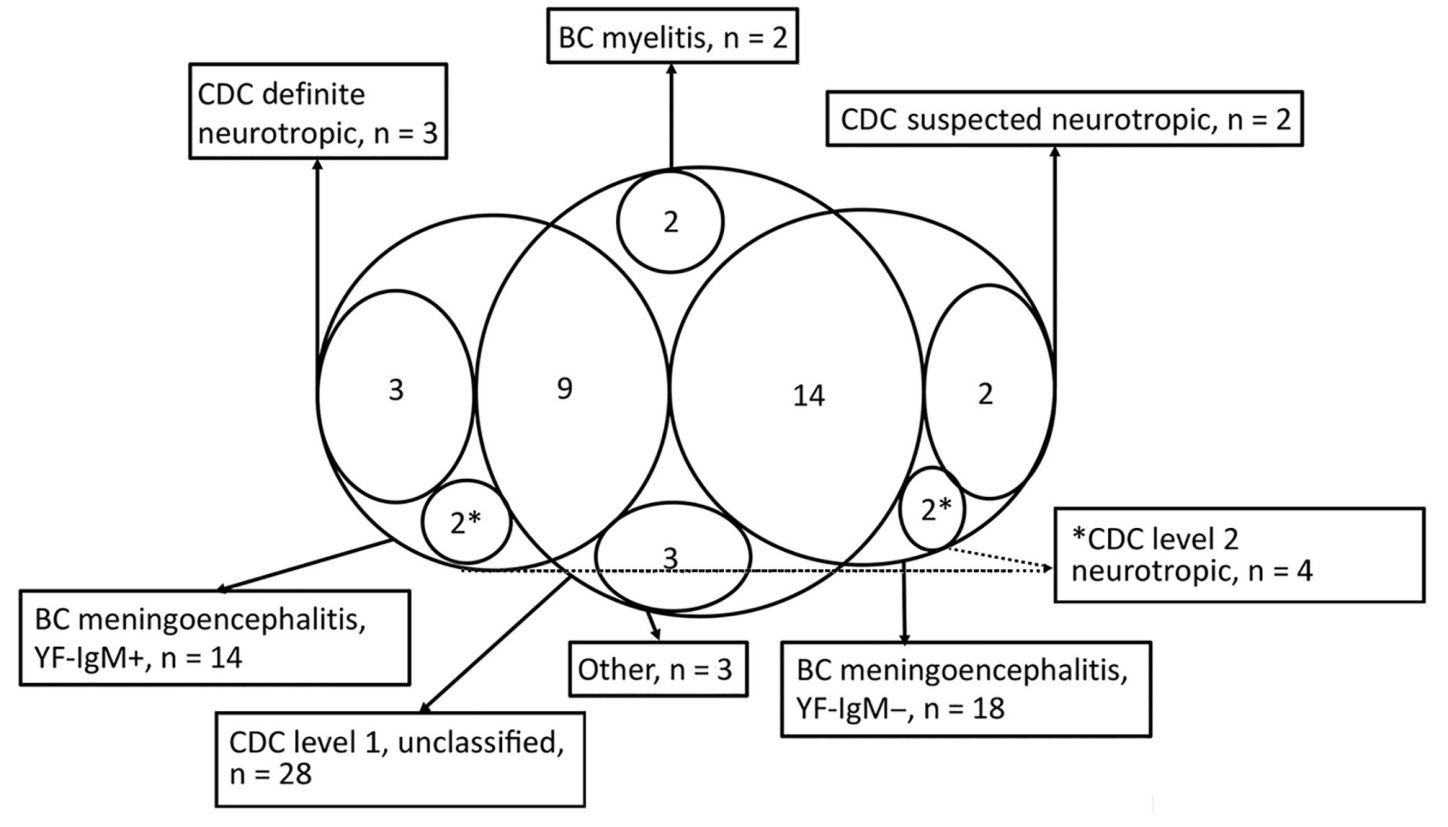
Classification of cases of yellow fever vaccine–associated neurologic disease, São Paulo, Brazil, 2017–2018. Excluded cases, acute disseminated encephalomyelitis cases, and Guillain-Barré syndrome cases not shown. The area with n= 9 represents the intersection between the group "BC meningoencephalitis, YF-IgM+ (reactive CSF-YF-IgM)” and “CDC level 1, unclassified.” The area with n = 14 represents the intersection between the group "BC meningoencephalitis, YF-IgM– (nonconfirmed)" and "CDC level 1, unclassified." BC, Brighton Collaboration criteria; CDC, Centers for Disease Control and Prevention criteria; level 1 unclassified, level 1 neurologic disease not classifiable as level 2; level 2 neurotropic, level 2 neurotropic disease not further classified as suspected or definite neurotropic disease; other, includes atypical yellow fever vaccine–associated neurologic disease (optic neuritis, n = 1; ataxia, n = 1; opsoclonus-myoclonus-ataxia syndrome, n = 1); +, positive.

The BC (Figure 1) and CDC (Figure 2) criteria differed in several respects (Figure 3). Of 8 patients in the encephalitis group, 3 were classified as having suspected or definite neurotropic disease according to the CDC criteria. Two cases of encephalitis could not be considered neurotropic disease because EEG and MRI were not performed, and for 3 novel autoimmune encephalitis cases, it was not possible to ascertain causality. The CDC criteria were particularly less inclusive of aseptic meningitis. Of the 24 patients with aseptic meningitis, only 2 were classified as having suspected or definite neurotropic disease (1 patient with meningeal enhancement on MRI, 1 with disorganized backgrounds on EEGs); 21 fell into the level 1 neurologic disease group (including 1 patient whose vaccine–symptom interval was 34 days), either for the absence of a typical MRI (unremarkable, 2 cases; not performed, 19 cases) or EEG findings (unremarkable, 2 cases; not performed, 19 cases). One case was classified as level 2 neurotropic disease but not further classified as suspected or definite neurotropic disease for a 38-day vaccine–symptom interval.

The CDC and BC criteria generally agreed on the classification of ADEM and GBS cases; only 1 GBS case was disregarded as YEL-AND by the CDC criteria because of symptom onset 31 days after vaccination. As expected, myelitis and unclassified cases were also missed by the CDC criteria.

## Discussion

Publications on AEFI with YF vaccine are limited mainly to case reports and small case series with varying case definitions. Most published cases do not meet the CDC or BC criteria (*15*). Some retrospective studies used different diagnostic criteria to evaluate incidence of YEL-AND during mass vaccination campaigns (*16*) or long periods of observation in specific regions (*3*,*4*,*6*,*17*). McMahon et al. described 15 cases that had been notified to the Vaccine Adverse Event Reporting System (https://vaers.hhs.gov) in a 15-year period; the criteria used differed slightly from the current CDC criteria: patients with level 1 neurologic disease were classified as having encephalitis, depending on the timing of symptoms or detection of YF IgM in CSF, regardless of MRI or EEG findings (*3*). A group in France used the same criteria but highlighted the differences between encephalitis and meningitis in their report of 4 patients with YEL-AND (*17*). In an active surveillance study during vaccination campaigns in Africa, Breugelmans et al. evaluated 164 suspected cases of severe AEFI, of which only 6 were considered YEL-AND according to the BC case definitions. YF virus IgM in CSF was assessed for only 2 patients, and results for both were negative (*16*). In a series of cases reported to the Vaccine Adverse Event Reporting System during 2007–2013, AEFI cases were classified as YEL-AND if they met the BC case definitions. A total of 17 events were included: 6 GBS, 6 aseptic meningitis, 2 encephalitis, 2 myelitis, and 1 ADEM (*6*). Detecting YF virus RNA in CSF samples is exceedingly rare, which was confirmed in our study.

Cross-reactivity with other flaviviruses was ruled out with DENV immunology. DENV is the main arbovirus in the state of São Paulo; in 2018, infection incidence was 43.4 cases/100,000 inhabitants. The combined incidence of Zika and chikungunya virus infections during the same period was <3 cases/100,000 inhabitants (*18*). All 32 CSF samples tested for DENV IgM were negative, which makes cross-reactivity with flaviviruses unlikely. The patients were evaluated at tertiary referral centers, which enabled a detailed analysis of the clinical characteristics of individual cases. This detailed analysis may be associated with the large proportion of cases with a high or intermediate level of diagnostic certainty according to the BC criteria. On the other hand, hospital-based retrospective studies may be inappropriate for estimating the incidence of events among vaccinees in the general population.

Comparing the case classifications obtained from each criterion revealed major limitations for those from CDC. The definition of neurotropic disease, which requires evidence suggestive of encephalitis on EEG or MRI scans, leads to many meningoencephalitis cases not being properly classified as level 2 neurologic disease, especially for patients with aseptic meningitis. This limitation is relevant for 2 reasons. First, many mass vaccination campaigns take place in countries where YF is endemic, notably low-income and lower-middle-income countries, where access to diagnostic tests is limited. Second, aseptic meningitis may be more common than encephalitis, as observed in our study and previously (*6*) and is typically devoid of parenchymal brain abnormalities that would be evident on an EEG or MRI scans. On the other hand, detection of pathogen-specific IgM-class antibodies in CSF is widely recognized as indicative of CNS viral invasion and constitutes a relevant indication of causality in the evaluation of suspected YEL-AND. We were able to diagnose meningoencephalitis in more cases by using the BC criteria to ascertain aseptic meningitis and encephalitis with reactive IgM in CSF as evidence of causality (14 cases) than by strictly applying the CDC criteria (3 cases). Increased sensitivity of the BC criteria was also reported by Lindsey et al. Of the 17 cases classified as YEL-AND by using BC-based criteria, only 13 were classified as such by using the CDC case definitions (*6*).

Another limitation of the CDC criteria is exclusion of vaccination as the cause for patients with a vaccine–symptom interval >30-days, which led to exclusion of 3 cases in our study, including 1 patient with reactive IgM in CSF in whom meningoencephalitis developed 38 days after vaccination. Similarly, modifying the CDC criteria enabled Martins et al. to include 2 patients with meningoencephalitis 39 and 36 days after vaccination (*4*).

Our study also expands the range of neurologic complications attributable to YF vaccine. We found 1 case of aseptic meningitis in a breast-feeding infant, which is very rare (only 3 cases with IgM in the CSF of breast-feeding infants have been reported to date [*19*–*21*]), and 3 cases of immune-mediated encephalitides (3 neuronal surface antibody encephalitides and 1 case of antibody-negative opsoclonus-myoclonus-ataxia), which are not traditionally associated with the YF vaccine. However, anti-NMDA-r encephalitis is triggered by infections (*22*), several other vaccines (*23*–*27*), and the YF vaccine (*28*,*29*); as such, anti-NMDA-r encephalitis could represent a novel YEL-AND. Opsoclonus-myoclonus-ataxia is considered a paraneoplastic or parainfectious disease, which is associated with several infections (*30*–*33*), other vaccines (*34*–*39*), and 1 case of YEL-AND described by Martins et al. (*4*).

Last, of the 39 patients with suspected YEL-AND for which information on vaccine dosing was available, 9 had received fractional doses and 30 had received full doses. Although the proportion of cases associated with fractional doses (1:3.3) is smaller than the proportion of fractional doses in the São Paulo region (1:1.5), this finding must be interpreted with caution because the participating centers of our study are tertiary referral centers with statewide catchment areas. AEFI surveillance data from Center for Epidemiologic Surveillance of the State of São Paulo did not show substantially different reporting rates between the 2 doses of the vaccine (E. Gatti Fernandes, unpub. data).

In conclusion, both full and fractional doses of the YF vaccine can cause YEL-AND. Aseptic meningitis is a YEL-AND for which the CDC criteria are particularly exclusive. In contrast to detecting YF virus IgM in CSF, limited value for diagnosing meningoencephalitis has been found for molecular testing, MRI, and EEG. Future studies of YEL-AND should be based on BC case definitions for case ascertainment and on detection of YF virus IgM in CSF for determination of causality for patients with aseptic meningitis and encephalitis. Autoimmune encephalopathies should be included as potential YEL-ANDs.

Appendix 1 Brighton Collaboration case definitions for yellow fever vaccine–associated neurologic disease.

Appendix 2 Centers for Disease Control and Prevention case ascertainment and case definitions for yellow fever vaccine–associated neurologic disease.

Appendix 3 Detailed information on individual cases of yellow fever vaccine–associated neurologic disease, São Paulo, Brazil, 2017–2018.
