# Supplementary material for: Neurologic Disease after Yellow Fever Vaccination, São Paulo, Brazil, 2017–2018
Source: Emerg Infect Dis. 2021 Jun;27(6):1577–87. doi: 10.3201/eid2706.204170 (PMC8153874; doi:10.3201/eid2706.204170)
Supplement: Appendix 1 — Brighton Collaboration case definitions for yellow fever vaccine–associated neurologic disease. [file 20-4170-Techapp-s1.pdf]

Article DOI: https://doi.org/10.3201/eid2706.204170

Neurologic Disease after Yellow Fever Vaccination, São Paulo, Brazil, 2017–2018

Appendix 1

Case definitions: aseptic meningitis (*1*), encephalitis, acute disseminated encephalomyelitis (ADEM) (*2*), myelitis (*2*), and Guillain-Barré syndrome (*3*) and assessment of causality in aseptic meningitis (*1*)

Case definition—aseptic meningitis

Level 1 of diagnostic certainty

- Clinical evidence of acute meningitis such as fever, headache, vomiting, bulging fontanelle, nuchal rigidity or other signs of meningeal irritation, AND
- Pleocytosis in CSF[a] determined as:
  - >5 leukocytes/mm^3 (L) if patient is 2 months of age[b] or older,
  - >15 leukocytes/mm^3 (L) in infants younger than 2 months,[b] AND
- Absence of any microorganism on Gram stain of CSF, AND
- Negative routine bacterial culture of CSF in the absence of antibiotic treatment before obtaining the first CSF sample.

Level 2 of diagnostic certainty

- Clinical evidence of acute meningitis such as fever, headache, vomiting, bulging fontanelle, nuchal rigidity or other signs of meningeal irritation, AND
- Pleocytosis in CSF[a] determined as:
  - >5 leukocytes/mm^3 (L) if patient is 2 months of age or older,

◦ >15 leukocytes/mm^3 (L) in infants younger than 2 months, AND

- Absence of any microorganism on Gram stain of CSF, AND
- No bacterial culture of CSF obtained, OR negative culture in the presence of antibiotic treatment before obtaining the first CSF sample.

Level 3 of diagnostic certainty

Not applicable If the case meets criteria for aseptic meningitis and encephalitis case definition, it should be reported only as encephalitis.

Case classification of aseptic meningitis cases for evaluation of aseptic meningitis as an adverse event following immunization

Confirmed vaccine-associated aetiology

- Identification of vaccine virus species in CSF by tissue- culture isolation or by PCR and sequencing or RFLP analysis confirms that virus is derived from a vaccine strain.

Probable vaccine-associated aetiology. All of the following:

- Prior vaccination or exposure to a person vaccinated with a transmissible live virus vaccine, AND
- Identification of vaccine virus species in CSF but sequence or RFLP analysis of virus strain have not been performed or results are ambiguous, AND
- No known concurrent circulation of the wild type virus (not used in vaccine) in the community, AND
- No identification of other aetiologic agent in CSF.

Possible vaccine-associated aetiology

All of the following:

- Prior vaccination or exposure to a person vaccinated with a transmissible live viral vaccine, AND

- Identification of vaccine virus species in CSF but sequence or RFLP analysis of virus strain have not been performed or results are ambiguous, AND
- Concurrent circulation of the wild type virus (not used in vaccine) is known or cannot be excluded, AND
- No identification of other aetiologic agent in CSF.

Unknown aetiology

- No aetiologic agent has been identified in CSF.

Non-vaccine-associated aetiology

- Identification of other infectious agent with no evidence of presence of vaccine virus.
- If vaccine virus species is detected in CSF, this virus strain has to be confirmed to be wild type virus by RFLP analysis

Case definitions: Guillain–Barré syndrome

Level 1 of diagnostic certainty

- Bilateral AND flaccid weakness of the limbs AND
- Decreased or absent deep tendon reflexes in weak limbs AND
- Monophasic illness pattern AND interval between onset and nadir of weakness between 12 h and 28 days AND

subsequent clinical plateau AND

- Electrophysiologic findings consistent with GBS[12] AND
- Cytoalbuminologic dissociation (i.e., elevation of CSF protein level above laboratory normal value AND CSF total white cell count <50 cells/ l)[13] AND
- Absence of an identified alternative diagnosis for weakness.

Level 2 of diagnostic certainty

- Bilateral AND flaccid weakness of the limbs AND

• Decreased or absent deep tendon reflexes in weak limbs AND

• Monophasic illness pattern AND

interval between onset and nadir of weakness between 12 h and 28 days AND

subsequent clinical plateau AND

CSF total white cell count <50 cells/ l (with or without CSF pro- tein elevation above laboratory normal value) OR

• IF CSF not collected or results not available, electrophysiologic studies consistent with GBS AND

• Absence of identified alternative diagnosis for weakness

Level 3 of diagnostic certainty

• Bilateral AND flaccid weakness of the limbs AND

• Decreased or absent deep tendon reflexes in weak limbs AND

• Monophasic illness pattern AND

• interval between onset and nadir of weakness between 12 h and 28 days AND subsequent clinical plateau

AND

Absence of identified alternative diagnosis for weakness

Case definition: Encephalitis

Level 1 of diagnostic certainty

(a) Demonstration of acute inflammation of central nervous system parenchyma (± meninges) by histopathology.

Level 2 of diagnostic certainty

(a) Encephalopathy (e.g. depressed or altered level of consciousness, lethargy, or personality change lasting >24 h), **AND INCLUDING**

(b) **ONE OR MORE** of the following:

1. Decreased or absent response to environment, as defined by response to loud noise or painful stimuli

2. Decreased or absent eye contact

3. Inconsistent or absent response to external stimuli,

4. Decreased arousability,

5. Seizure associated with loss of consciousness

OR

(c) Focal or multifocal findings referable to the central nervous system, including one or more of the following:

1. Focal cortical signs (including but not limited to: aphasia, alexia, agraphia, cortical blindness)

2. Cranial nerve abnormality/abnormalities

3. Visual field defect/defect(s)

4. Presence of primitive reflexes (Babinski's sign, glabellar reflex, snout/sucking reflex)

5. Motor weakness (either diffuse or focal; more often focal)

6. Sensory abnormalities (either positive or negative; sensory level),

7. Altered deep tendon reflexes (hypo- or hyperreflexia, reflex asymmetry),

8. Cerebellar dysfunction, including ataxia, dysmetria, cerebellar nystagmus.

AND (for both possibilities to reach Level 2)

(d) **TWO OR MORE** of the following indicators of inflammation of the CNS:

1. Fever (temperature $\geq 38 \circ C$),

2. CSF pleocytosis (>5WBC/mm3 in children >2 months of age; >15 WBC/mm3 in children <2 months of age),

3. EEG findings consistent with encephalitis

4. Neuroimaging consistent with encephalitis.

Level 3 of diagnostic certainty

(a) Encephalopathy (e.g. depressed or altered level of consciousness, lethargy, or personality change lasting >24 h), **AND INCLUDING**

(b) **ONE OR MORE** of the following:

1. Decreased or absent response to environment, as defined by response to loud noise or painful stimuli

2. Decreased or absent eye contact

3. Inconsistent or absent response to external stimuli,

4. Decreased arousability,

5. Seizure associated with loss of consciousness

OR

(c) Focal or multifocal findings referable to the central nervous system, including one or more of the following:

1. Focal cortical signs (including but not limited to: aphasia, alexia, agraphia, cortical blindness)

2. Cranial nerve abnormality/abnormalities

3. Visual field defect/defect(s)

4. Presence of primitive reflexes (Babinski's sign, glabellar reflex, snout/sucking reflex)

5. Motor weakness (either diffuse or focal; more often focal)

6. Sensory abnormalities (either positive or negative; sensory level),

7. Altered deep tendon reflexes (hypo- or hyperreflexia, reflex asymmetry),

8. Cerebellar dysfunction, including ataxia, dysmetria, cerebellar nystagmus.

AND (for both possibilities to reach Level 3)

(d) **ONE** of the following indicators of inflammation of the CNS:

1. Fever (temperature ≥38 ◦ C),
2. CSF pleocytosis (>5WBC/mm3 in children >2months of age; >15 WBC/mm3 in children <2 months of age),
3. EEG findings consistent with encephalitis
4. Neuroimaging consistent with encephalitis.

Case definition: myelitis

Level 1 of diagnostic certainty

(a) Demonstration of acute spinal cord (± meninges) by histopathology.

Level 2 of diagnostic certainty

(a) Myelopathy (development of sensory, motor, or autonomic dysfunction attributable to the spinal cord, including upper- and/or lower-motor neuron weakness, sensory level, bowel and/or bladder dysfunction, erectile dysfunction)

AND

(b) **TWO OR MORE** of the following indicators suggestive of spinal cord inflammation:

1. Fever (temperature>=38oC),
2. CSF pleocytosis (>5 WBC/mm^3 in children>2months of age; >15WBC/mm^3 in children<2 months of age,
3. Neuroimaging findings demonstrating acute inflammation (+-meninges, or demyelination of the spinal cord.

OR

(c) Focal or multifocal findings referable to the central nervous system, including one or more of the following:

1. Focal cortical signs (including but not limited to: aphasia, alexia, agraphia, cortical blindness)
2. Cranial nerve abnormality/abnormalities

3. Visual field defect/defect(s)

4. Presence of primitive reflexes (Babinski's sign, glabellar reflex, snout/sucking reflex)

5. Motor weakness (either diffuse or focal; more often focal)

6. Sensory abnormalities (either positive or negative; sensory level),

7. Altered deep tendon reflexes (hypo- or hyperreflexia, reflex asymmetry),

8. Cerebellar dysfunction, including ataxia, dysmetria, cerebellar nystagmus.

AND (for both possibilities to reach Level 2)

(d) **TWO OR MORE** of the following indicators of inflammation of the CNS:

1. Fever (temperature ≥38 ◦ C),

2. CSF pleocytosis (>5WBC/mm3 in children >2months of age; >15 WBC/mm3 in children <2 months of age),

3. EEG findings consistent with encephalitis

4. Neuroimaging consistent with encephalitis.

Level 3 of diagnostic certainty

(a) Myelopathy (development of sensory, motor, or autonomic dysfunction attributable to the spinal cord, including upper- and/or lower-motor neuron weakness, sensory level, bowel and/or bladder dysfunction, erectile dysfunction)

AND

(b) **ONE** of the following indicators suggestive of spinal cord inflammation:

1. Fever (temperature>=38oC),

2. CSF pleocytosis (>5 WBC/mm^3 in children>2months of age; >15WBC/mm^3 in children<2 months of age,

3. Neuroimaging findings demonstrating acute inflammation (+-meninges, or demyelination of the spinal cord.

Exclusion criterion for Levels 2 and 3 of diagnostic certainty

(a) Other diagnosis for illness present

Acute disseminated encephalomyelitis (ADEM) (2)

Level 1 of diagnostic certainty

(a) Demonstration of diffuse or multifocal areas of demyelination by histopathology.

OR

Focal or multifocal findings referable to the central nervous system, including one or more of the following:

1. Encephalopathy (see case definition for encephalitis or specification of encephalopathy),
2. Focal cortical signs (including but not limited to: aphasia, alexia, agraphia, cortical blindness),
3. Cranial nerve abnormality/abnormalities,
4. Visual field defect/defects,
5. Presence of primitive reflexes (Babinski's sign, glabellar reflex, snout/sucking reflex),
6. Motor weakness (either diffuse or focal; more often focal),
7. Sensory abnormalities (either positive or negative; sensory level),
8. Altered deep tendon reflexes (hypo- or hyperreflexia, asymmetry of reflexes), or.
9. Cerebellar dysfunction, including ataxia, dysmetria, cerebellar nystagmus,

AND

(c) Magnetic resonance imaging (MRI) findings displaying diffuse or multifocal white matter lesions on T2-weighted, diffusion-weighted (DWI) or fluid-attenuated inversion recovery (FLAIR) sequences (+- gadolinium enhancement on T1 sequences)

AND

Monophasic illness (i.e., absence of relapse within a minimum of 3 months of symptomatic nadir)

Level 2 of diagnostic certainty

(a) Focal or multifocal findings referable to the central nervous system, including one or more of the following:

10. Encephalopathy (see case definition for encephalitis for specification of encephalopathy),

11. Focal cortical signs (including but not limited to: aphasia, alexia, agraphia, cortical blindness),12. Cranial nerve abnormality/abnormalities,

12. Cranial nerve abnormalities,

13. Visual field defect/defects,

14. Presence of primitive reflexes (Babinski's sign, glabellar reflex, snout/sucking reflex),

15. Motor weakness (either diffuse or focal; more often focal),

16. Sensory abnormalities (either positive or negative sensory level),

17. Altered deep tendon reflexes (hypo-or hyperreflexia, asymmetry of reflexes), or

18. Cerebellar dysfunction, including ataxia, dysmetria, cerebellar nystagmus

AND

(c) Magnetic resonance imaging (MRI) findings displaying diffuse or multifocal white matter lesions on T2-weighted, diffusion-weighted (DWI) or fluid-attenuated inversion recovery (FLAIR) sequences (+- gadolinium enhancement on T1 sequences)

AND

Insufficient follow up time achieved to document absence of relapse within a minimum period of 3 months following symptomatic nadir

Level 3 of diagnostic certainty

(a) Focal or multifocal findings referable to the central nervous system, including one or more of the following:

19. Encephalopathy (see case definition for encephalitis for specification of encephalopathy),

20. Focal cortical signs (including but not limited to: aphasia, alexia, agraphia, cortical blindness),12. Cranial nerve abnormality/abnormalities,

21. Cranial nerve abnormalities

22. Visual field defect/defects,

23. Presence of primitive reflexes (Babinski's sign, glabellar reflex, snout/sucking reflex),

24. Motor weakness (either diffuse or focal; more often focal),

25. Sensory abnormalities (either positive or negative sensory level),

26. Altered deep tendon reflexes (hypo-or hyperreflexia, asymmetry of reflexes), or

27. Cerebellar dysfunction, including ataxia, dysmetria, cerebellar nystagmus

References

1. Tapiainen T, Prevots R, Izurieta HS, Abramson J, Bilynsky R, Bonhoeffer J, et al.; Brighton Collaboration Aseptic Meningitis Working Group. Aseptic meningitis: case definition and guidelines for collection, analysis and presentation of immunization safety data. Vaccine. 2007;25:5793–802. PubMed https://doi.org/10.1016/j.vaccine.2007.04.058

2. Sejvar JJ, Kohl KS, Bilynsky R, Blumberg D, Cvetkovich T, Galama J, et al.; Brighton Collaboration Encephalitis Working Group. Encephalitis, myelitis, and acute disseminated encephalomyelitis (ADEM): case definitions and guidelines for collection, analysis, and presentation of immunization safety data. Vaccine. 2007;25:5771–92. PubMed https://doi.org/10.1016/j.vaccine.2007.04.060

3. Sejvar JJ, Kohl KS, Gidudu J, Amato A, Bakshi N, Baxter R, et al.; Brighton Collaboration GBS Working Group. Guillain-Barré syndrome and Fisher syndrome: case definitions and guidelines for collection, analysis, and presentation of immunization safety data. Vaccine. 2011;29:599–612. PubMed https://doi.org/10.1016/j.vaccine.2010.06.003
