# Supplementary material for: Neurologic Disease after Yellow Fever Vaccination, São Paulo, Brazil, 2017–2018
Source: Emerg Infect Dis. 2021 Jun;27(6):1577–87. doi: 10.3201/eid2706.204170 (PMC8153874; doi:10.3201/eid2706.204170)
Supplement: Appendix 2 — Centers for Disease Control and Prevention case ascertainment and case definitions for yellow fever vaccine–associated neurologic disease. [file 20-4170-Techapp-s2.pdf]

Article DOI: https://doi.org/10.3201/eid2706.204170

Neurologic Disease after Yellow Fever Vaccination, São Paulo, Brazil, 2017–2018

Appendix 2

A

Case ascertainment

Level 1: neurologic disease

- One or more of the following signs and symptoms:
  - -- fever (≥100.5°F [>38.1°C] for >24 hours) and headache (>24 hours)
  - -- focal neurologic dysfunction (including but not limited to ataxia, aphasia, and paresis)
  - -- mental status change (confusion, lethargy, or personality change lasting >24 hours)
  - -- new onset seizure or recurrence of previously controlled seizures
  - -- cerebrospinal fluid (CSF) pleocytosis (>5 WBC/mm^3)
  - -- elevated CSF protein (>1.5 times the normal limit)

Level 2: neurotropic disease

- Level 1 neurologic disease, and
- one or more of the following:
  - -- neuroimaging consistent with inflammation, (with or without demyelination)
  - -- electroencephalogram finding consistent with encephalopathy

Level 2: autoimmune disease with central nervous system involvement

- Level 1 neurologic disease, and
- neuroimaging consistent with multifocal or disseminated areas of demyelination

Level 2: autoimmune disease with peripheral nervous system involvement

- Level 1 neurologic disease,[†] and
- two or more of the following signs and symptoms:
  - -- limb weakness with decreased or absent tendon reflexes
  - -- cranial nerve abnormalities
  - -- autonomic dysfunction (including but not limited to postural hypotension, arrhythmias, abnormal sweating, and gastric motility abnormalities)
  - -- numbness or paresthesias in the extremities
  - -- electromyography finding consistent with Guillain-Barré syndrome

B

Case definitions

Yellow fever vaccine-associated neurotropic disease

Suspect neurotropic disease

- Onset of symptoms and signs occurs within 1--30 days of vaccination with yellow fever vaccine, either given alone or in combination with other vaccinations;
- level 2 neurotropic disease; and
- no evidence of other diagnoses

Probable neurotropic disease

- Suspect YEL-AND, and
- one or more of the following:
  -- vaccine-type yellow fever viral isolation from blood (>7 days postvaccination)
  -- yellow fever 17D[§] virus concentration in serum on any day exceeds 3 $\log_{10}$ pfu/mL

Definite neurotropic disease

- Suspect YEL-AND, and
- one or more of the following:
  -- YF-specific CSF IgM
  -- yellow fever 17D[§] virus isolation from CSF
  -- amplification of vaccine type virus[§] from CSF

Yellow fever vaccine-associated autoimmune disease with central nervous system involvement (YEL-AAD-CNS)

Suspect YEL-AAD-CNS

- Onset of symptoms and signs occurs within 1--30 days of vaccination with yellow fever vaccine, either given alone or in combination with other vaccinations;
- level 2 autoimmune disease with central nervous system involvement; and
- no evidence of other diagnoses

Probable YEL-AAD-CNS

- Suspect autoimmune disease with central nervous system involvement, and
- YEL administered alone

Yellow fever vaccine-associated autoimmune disease with peripheral nervous system involvement (YEL-AAD-PNS)

Suspect YEL-AAD-PNS

- Onset of symptoms and signs occurs within 1--30 days of vaccination with yellow fever vaccine, either given alone or in combination with other vaccinations;
- level 2 autoimmune disease with PNS involvement; and
- no evidence of other diagnoses

Probable YEL-AAD-PNS

- Suspect autoimmune disease with PNS involvement, and
- YEL administered alone

Figure. Case ascertainment (A) and CDC's Yellow Fever Vaccine Safety (YFVS) Working Group Case Definition for yellow fever–vaccine associated neurologic disease (YEL-AND) (B). Data from Staples JE, Gershman M, Fischer M; Centers for Disease Control and Prevention (CDC). Yellow fever vaccine: recommendations of the Advisory Committee on Immunization Practices (ACIP). MMWR Recomm Rep. 2010;59(RR-7):1–27.
